# Cost of digital technologies and family-observed DOT for a shorter MDR-TB regimen: a modelling study in Ethiopia, India and Uganda

**DOI:** 10.1186/s12913-023-10295-z

**Published:** 2023-11-18

**Authors:** Laura Rosu, Jason Madan, Gay Bronson, Jasper Nidoi, Mamo G. Tefera, Muniyandi Malaisamy, Bertel S. Squire, Eve Worrall

**Affiliations:** 1 Clinical Sciences Department, Liverpool School of Tropical Medicine, Liverpool, UK; 2 Warwick Medical School, University of Warwick, Coventry, UK; 3 Vital Strategies, New York, NY USA; 4 Makerere University Lung Institute, Kampala, Uganda; 5 Addis Ababa Science and Technology University, Addis Ababa, Ethiopia; 6 National Institute for Research in Tuberculosis, Chennai, India; 7 Liverpool School of Tropical Medicine, Vector Biology Department, Liverpool, UK

**Keywords:** Tuberculosis, Digital technology, DOT, MDR-TB, Shorter regimen, Cost

## Abstract

**Background:**

In 2017, the WHO recommended the use of digital technologies, such as medication monitors and video observed treatment (VOT), for directly observed treatment (DOT) of drug-susceptible TB. The WHO’s 2020 guidelines extended these recommendations to multidrug-resistant tuberculosis (MDR-TB), based on low evidence. The impact of COVID on health systems and patients underscored the need to use digital technologies in the management of MDR-TB.

**Methods:**

A decision-tree model was developed to explore the costs of several potential DOT alternatives: VOT, 99DOTS (Directly-observed Treatment, Short-course) and family-observed DOT. Assuming a 9-month, all-oral regimen (as evaluated within the STREAM trial), we constructed base-case cost models for the standard-of-care DOTs in Ethiopia, India, and Uganda, as well as for the three alternative DOT approaches. The models were populated with STREAM Stage 2 clinical trial outcome and cost data, supplemented with market prices data for the digital DOT strategies. Sensitivity analyses were conducted on key parameters.

**Results:**

Modelling suggested that the standard-of-care DOT approach is the most expensive DOT strategy from a societal perspective in all three countries evaluated (Ethiopia, India, Uganda), with considerable direct- and indirect-costs incurred by patients. The second most expensive DOT approach is VOT, with high health-system costs, largely caused by up-front technology expenditure.

Each of VOT, 99DOTS and family-observed DOT would reduce by more than 90% patients’ direct and indirect costs compared to standard of care DOT.

Results were robust to the sensitivity analyses.

**Conclusions:**

While data on the costs and efficacy of alternative DOT approaches in the context of shorter MDR-TB treatment is limited, our modelling suggests alternative DOT approaches can significantly reduce patient costs in all three countries. Health system costs are higher for VOT and lower for 99DOTS and family-observed therapy when compared to standard of care DOT, as low smartphone penetration and internet availability requires the VOT health system to fund the cost of making them available to patients.

**Supplementary Information:**

The online version contains supplementary material available at 10.1186/s12913-023-10295-z.

## Background

Tuberculosis (TB) is a disease caused by bacteria that are spread through air. Multi-drug resistant tuberculosis (MDR-TB) is caused by strains of TB bacteria that do not respond to the two most potent anti-TB drugs [1]. In 2019, at the global level, half a million people developed rifampicin-resistant TB (RR-TB), and 78% of these had MDR-TB [1]. The WHO End TB strategy [2] aims to end the global TB epidemic by 2035 and, amongst other targets, it aimed to reduce the percentage of affected families facing catastrophic TB costs to zero by 2020. However, most countries did not reach this milestone. Additionally, the COVID-19 pandemic reversed progress made towards global TB targets, demanding a renewed focus on improving access to acceptable treatments and treatment success rates.

Globally, 2019 treatment success rates for drug-susceptible TB were 86% but only 60% for MDR-TB, with more than 15% of unfavourable results attributable to patients who were lost to follow-up [1]. For many years, the recommended treatment for MDR-TB included injectable agents and lasted as long as 20 months. In 2020, the WHO recommended a new shorter, all-oral (9–11 months) regimen for patients with MDR-TB and more recently a 6-month all-oral regimen [3, 4]. However, the 9-month all-oral regimen is still in widespread use. Research has shown that patients find it easier to complete shorter all oral regimens, compared with previously recommended injectable-containing longer regimens [3].

Directly Observed Treatment, Short-course (DOTS) strategy has been recommended by the WHO since 1993. It has been a successful approach to TB control in many countries. Traditionally, in-person observation of patient treatment adherence by health professionals (SOC DOT) was a key component of the DOTs strategy [5]. In 2017, to address patient and health system needs, however, the WHO Global TB Programme formulated new recommendations for DOT of drug-susceptible TB (DS-TB) [6] to make it more patient-centred. Key aspects of the updated guidelines recommend the use of electronic and mobile phone applications, known as digital health interventions. These have been used successfully to improve treatment adherence in the context of HIV and NCDs [7, 8], and can include use of Short Message Services (SMS) or phone calls for medication reminders, medication monitors, and video-observed treatment (VOT). The 2020 MDR-TB guidelines extended the digital intervention recommendations to MDR-TB, acknowledging their potential contribution to making MDR-TB management more patient-centred [9]; however, the 2020 recommendations rated the certainty of evidence supporting the use of digital interventions to support adherence as very low. A WHO review of community contributions to TB care and recommendations to national TB programmes mentions that family members can act as DOT supervisors [10].

There is some evidence that VOT and MM can achieve similar treatment completion rates as SOC DOT in patients being treated for DS-TB, with similar numbers of missed doses. There is also limited evidence that family-observed DOT can achieve similar treatment success in MDR-TB patients who received the longer 20–24 month regimen. However, the cost, cost-effectiveness and effect on adherence and clinical outcomes of these interventions in the context of shorter MDR-TB regimens are unknown (see supplement).

There is however some evidence that digital health interventions can improve treatment adherence in people with drug-susceptible TB; however, no effect on clinical outcomes (cure, failure, death) has been observed [11].

This paper evaluates the cost of the three of the most used alternatives to SOC DOT- VOT, 99DOTS (a real time remote monitoring of intake of TB treatment using low-cost mobile phone-based technology) and family-observed DOT- for patients receiving a 9-month, all-oral MDR-TB treatment as tested in STREAM Stage 2 and that is similar to the WHO recommended regimen in 2020. It is thought these interventions enhance the patient’s autonomy, while still enabling health workers to monitor treatment adherence. Moreover, due to the longer duration of MDR-TB treatment and considerably higher costs of treatment borne by MDR-TB patients compared to DS-TB patients [12], the potential benefits to MDR-TB patients of alternative DOT approaches are likely to be even greater than for drug-susceptible TB.

## Methods

### Study setting

Ethiopia, India and Uganda are three of the 30 high TB burden countries, with an MDR/RR-TB incidence, in 2021, of 1.5 cases (95% CI 0.9- 2.1) per 100,000 population, 8.5 (95% CI 6.6- 10.0) and 3.2 (95% CI 1.0- 5.5), respectively [1, 13]. All three countries use a bedaquiline-based 9-month all-oral regimen similar to the STREAM 2 regimen as their standard of care for MDR-TB, and had STREAM Stage 2 study sites. STREAM was the largest recruited clinical trial to examine shortened regimens for MDR-TB.

In all three countries, most MDR-TB patients initiate treatment for MDR-TB at a TB hospital as outpatients and their treatment is then monitored by the district TB programs. Outpatient treatment is typically delivered using SOC DOT, meaning that MDR-TB patients travel daily in Ethiopia and Uganda and three times a week in India, to district health centres where they receive and take their TB medication. Usually, these district health centres are not fully decentralised to the patient’s community, so patients will incur out-of-pocket expenses for transport and/or food [14, 15] and income loss to take their treatment. This can have a substantial cost for patients, impact other competing activities in a patient’s life (opportunity cost) and also lead to missed doses or loss to follow-up (LTFU) [16].

### Description of interventions

In this study we evaluate VOT, 99DOTs and family-observed DOT compared to SOC DOT. These interventions were selected based on a 2018 systematic review [17] which showed that VOT and medication monitoring (MM) achieved similar treatment completion rates as SOC DOT in patients being treated for DS-TB, with similar numbers of missed doses.

When access to technology is limited, family-observed DOT can be an alternative to digital DOT [10]. A study showed no statistically significant difference in terms of treatment success as compared to SOC DOT (Family-observed DOT: 72%, 95% CI: 31.5- 93.5%; SOC DOT: 65.8%, 95% CI 55.7- 74.7%) in MDR-TB patients receiving the longer (20–24 month) treatment [18]. Little or no difference was observed in cure or treatment completion rates.

#### VOT

VOT is a smartphone-based approach that allows for remote treatment monitoring through either live or patient-recorded videos.

Studies conducted in the US and UK [19, 20] for DS-TB reported higher adherence with VOT, including in vulnerable populations. However, in the US, the effect on treatment completion rates was not statistically significant [21]. VOT substantially reduced healthcare personnel time needed for DOT supervision in both studies.

#### 99DOTS

99DOTS employs a low-cost mobile phone-based technology that enables real-time remote medication monitoring [22]. The anti-TB drugs blister packs are wrapped in a custom envelope that, when dispensing pills reveals hidden phone numbers. Patients then use any phone to call the number revealed, at no cost. The call is automatically recorded in the patient’s file and used to track adherence.

A large randomised controlled trial [11] of treatment support for active, DS-TB conducted in China reported that MM had an effect on treatment adherence relative to SOC DOT, with 29.9% of doses missed in the SOC DOT arm versus 17.0% in the medication monitor arm. However, there was no demonstrated impact on clinical outcomes. Since 2018, this DOT approach has been widely used in India for DS-TB, with more than 200,000 patients enrolled [22]. Amongst its benefits are the greater convenience and reduced stigma for patients [23].

#### Family-observed DOT

Under family-observed DOT daily treatment is supervised by a household member or friend selected by the patient, with drugs provided to the family member supervisor every two weeks. This reduces the patient’s visits to the DOT facility and stigma associated with visiting the centre on a daily basis [24]. Randomised controlled trials showed that there was no significant difference between treatment success rates of SOC DOT versus family-observed DOT in DS-TB patients [25].

#### Description of SOC DOT

MDR-TB patients initiate treatment at a TB hospital and, after the intensive phase, their treatment is then monitored by the district TB programs. Health workers at the district TB programs then deliver and supervise treatment. To receive treatment, patients travel daily in Ethiopia and Uganda and three times a week in India, to the DOT facility, incurring both direct and indirect costs.

### Decision analytic model

**Fig. 1:**
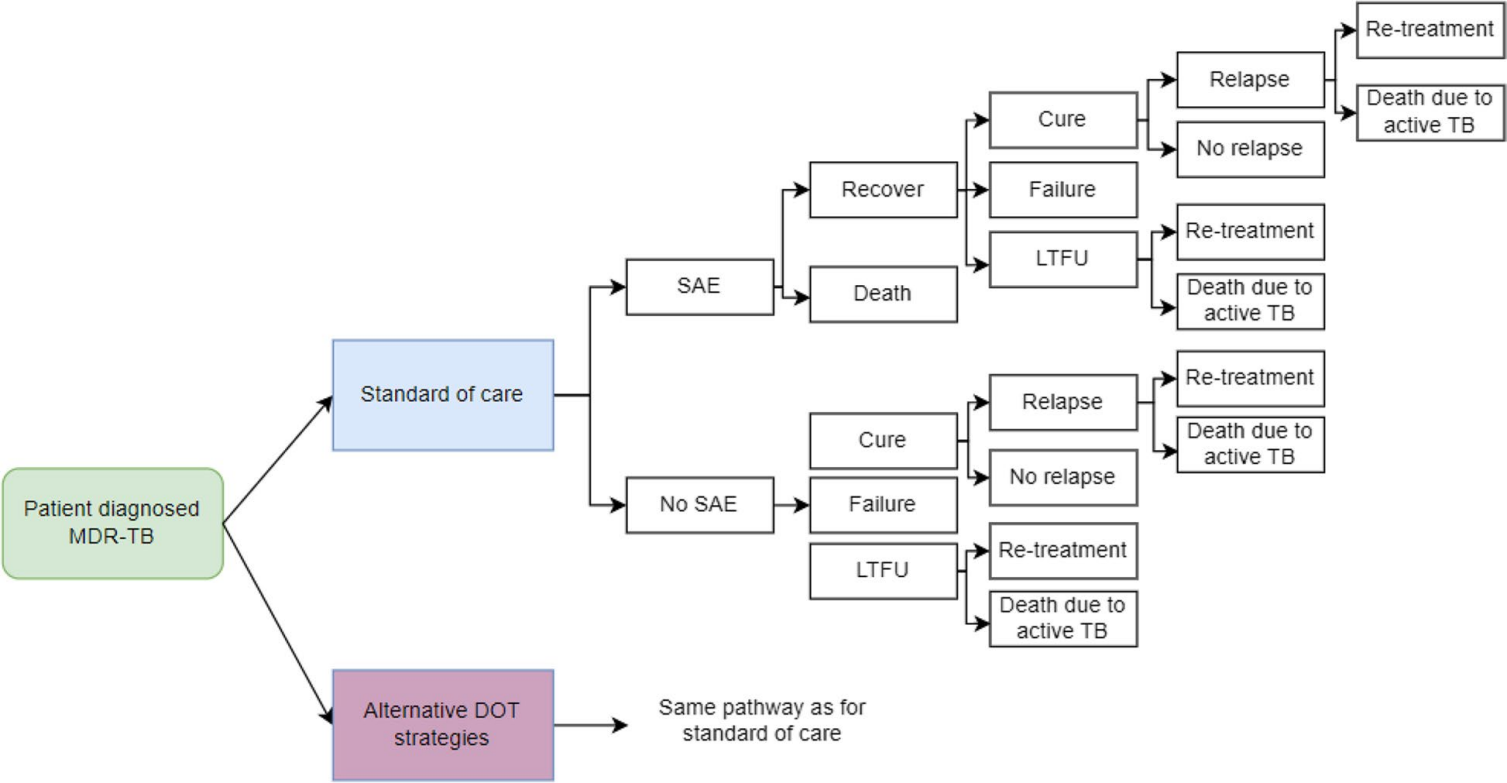
Visual representation of decision analytic model of standard of care and alternative DOT approaches. Source: Authors. Acronyms: SAE- Serious Adverse Event, LTFU- lost to follow-up. Final outcomes follow WHO categories: cured, failure, LTFU or death. Failure is defined as unfavourable outcomes as a result of treatment extension longer than 8 weeks after adverse event, or extension or change for other reasons, including adverse event, or consent withdrawal, lack of culture conversion and bacteriological reversion on treatment. Cure is defined as a treatment outcome that is not failure or LTFU. Relapse is defined as bacteriological reversion on treatment. Death was considered an SAE

A decision analytic model was developed in Excel (Fig. 1) to compare the costs of the above-mentioned DOT approaches in Ethiopia, India and Uganda. Costs were evaluated for patients receiving the 40-week, all-oral MDR-TB regimen, as evaluated in the STREAM Stage 2 trial, to construct the base-case standard of care DOT model in each country [26].

Several key assumptions were incorporated into the model. It was assumed that all DOT approaches yield the same cure, failure, LTFU and death rates. We made this assumption because there is no reported evidence regarding the impact of alternative DOT approaches on treatment outcomes for shorter MDR-TB regimens. It was assumed that patients enter the model once they start treatment and are treated as outpatients during the whole treatment period, as this reflects usual practice in all three countries. Patients who do not experience an SAE exit the model 40 weeks after treatment start if they are cured and experience no relapse, at 24 weeks if they are LTFU or die due to active TB, or at 80 weeks if they relapse and receive re-treatment. Patients who experience an SAE exit the model at 48 weeks after treatment start if they are cured and experience no relapse, at 32 weeks if they are LTFU or die due to active TB or at 80 weeks if they relapse and receive re-treatment. The reason patients who experience SAE exit the model later is because there is some evidence that SAEs result in treatment extension [27]; we therefore assumed that treatment can be extended by 8 weeks, the maximum period allowed in the trial before an outcome was categorised as unfavourable.

Total number of DOT visits for each strategy was 280 in Ethiopia and Uganda, and 120 in India (Table 1). For SOC DOT, those visits were in person; for the alternative DOT strategies, those “visits” were virtual or in person in the patient’s home (for family-observed DOT). In addition to DOT visits, in accordance with the 2022 operational handbook on tuberculosis, the model assumes patients travelled monthly to health facilities for in person clinical and safety monitoring, adding an additional nine in person visits to the DOT visits for each approach (see supplement for details on the tests done) [28].

**Table 1:** Number of visits for each of the DOT strategies

	Ethiopia, Uganda		India	
	**In person visits**	**Virtual/home visits**	**In person visits**	**Virtual/home visits**
SOC DOT	289	0	129	0
VOT	9	280	9	120
99DOTS	9	280	9	120
Family-observed DOT	9	280	9	120

Probability of different treatment outcomes and SAEs for the 9-month regimen were calculated based on the STREAM Stage 2 trial outcomes (Table 2) and were assumed to be the same across all countries [29].

**Table 2:** Probabilities used in the model, derived from the STREAM Stage 2 trial outcomes

Parameters	Probability
Probability of SAE	0.18
Probability of cure if no SAE	0.86
Probability of failure if no SAE	0.11
Probability LTFU if no SAE	0.03
Probability of recovering after SAE	0.82
Probability of death after SAE	0.18
Probability of relapse after cure after SAE	0.02
Probability of no relapse after cure after SAE	0.98
Probability of relapse after cure	0.02
Probability of no relapse after cure	0.98
Probability of cure after SAE	0.85
Probability of failure after SAE	0.12
Probability of LTFU after SAE	0.03

In addition to this, a 10% probability of death due to untreated active RR-TB after relapse was applied [1].

### Cost data

Main cost data source was STREAM Stage 2 trial data [26], supplemented by market prices or published estimates for costing alternative DOT strategies (see supplement).

### Health system costs

For costing VOT, we used market prices in each country in costing the smartphones and mobile data required. We assumed a 5-min appointment duration for each VOT visit [30]; for a video call of this duration, it was calculated that 500 MB of data per patient per month would be needed [31]. Monthly data usage was costed using in country data bundle costs. Smartphone penetration rates (more than 70% of Ugandans, 66% of Ethiopians and 57% of Indians did not own a smartphone in 2021) and internet usage data were used to calculate the percentage of population requiring a device and mobile data. To this, we added the costs related to the staff performing the monitoring activities for each strategy.

For costing 99DOTS, we included the per patient fixed cost of renting a toll-free line, the envelopes costs, SMS, call and staff packaging costs from manufacturer published data [22]. As for 99DOTS there is no need for a manned call, only costs related to healthcare worker training and adherence monitoring were included, assuming a 15-min duration per dose per patient.

For family-observed DOT costs, it was assumed that the family-member did not receive any pay for supervising their relative’s treatment. It was also assumed the family member was trained at the beginning of treatment and then every 12 weeks on how to monitor treatment adherence. Staff time of healthcare workers conducting that training was also included.

For SOC DOT, staff costs were calculated assuming a 15-min in-person visit duration.

Mean SAE costs from STREAM were added to the health system costs in each country. Also, costs related to monitoring tests and resources used during the in-person visits were also from STREAM (see supplement). The type of tests done and their quantities were taken from the 2022 operational handbook on tuberculosis.

### Patient costs

Both direct and indirect patient costs were included.

In terms of direct costs, we included the costs for attending DOT visits and monitoring visits, as reported by patients in the STREAM trial, up until week 40 of treatment. No costs related to post-treatment follow-up were included.

For calculating indirect costs, we used mean patient-reported income before MDR-TB diagnosis from STREAM to calculate cost per working minute. This cost was then multiplied by the visit durations for each DOT delivery strategy, assuming that the patient would be able to return to work after DOT.

Societal costs were calculated by summing total health system and patient costs.

### Sensitivity analyses

We conducted probabilistic sensitivity analysis to assess parameters uncertainty (see supplement) using 1000 Monte Carlo simulations. We fitted beta distributions for probabilities and gamma for costs. Where ranges were not available for costs, we used ± 30% as a range for mean costs.

The digital DOT and family observed DOT approaches are generally better accepted by patients, improving their commitment to treatment. This in turn can reduce the LTFU rates compared to SOC DOT. Therefore, we varied this parameter in a deterministic sensitivity analysis, by reducing the LTFU rate in the digital DOT and family observed DOT by 5% and 10%. However, DOT that is not supervised by a health worker might result in worse medication adherence, so in the sensitivity analysis we also tested a higher recurrence rate, by 6.5%, compared to the base case, for the alternative DOT strategies [32–34].

## Results

All base case results are in Table 3.

**Table 3:** Health system, patient and societal costs for each DOT strategy in each country, per patient

	Ethiopia (US$)			India (US$)			Uganda (US$)		
	Health system	Patient	Societal	Health system	Patient	Societal	Health system	Patient	Societal
SOC	3790.4	572.3	4362.6	2003.3	324.2	2327.4	6348.6	888.6	7237.1
VOT	3999.9	17.9	4017.8	2201.7	22.7	2224.4	6716.7	27.7	6744.5
99DOTS	3769.3	17.9	3787.2	1980.4	22.1	2002.5	6151.2	27.4	6178.7
Family-observed	3765.4	26.3	3791.7	2005.0	31.8	2036.7	5975.0	29.5	6004.4

### Patient costs

When compared to SOC DOT, adoption of VOT or 99DOTS reduces patient costs by 97% in Ethiopia and Uganda, and by 93% in India.

Although family-observed DOT is slightly more expensive than VOT and 99DOTS in all countries due to the monitoring training required, it would still save patients over 90% of costs in all countries when compared to SOC DOT.

### Health system costs

From a health system perspective, VOT was the most expensive DOT strategy, with a cost increase ranging from 5% in Uganda to 10% in India when compared to SOC. Higher health-system costs for VOT were primarily driven by up-front technology expenditure to purchase smartphones for patients because of low smartphone penetration rates.

Health system costs for the 99DOTS were slightly lower than SOC in all three countries, with savings ranging from 1% in Ethiopia and India to 3% in Uganda. This is due to a slight reduction in staff costs, as 99DOTS requires reduced staff contact time.

With respect to health system costs, family-observed DOT was the cheapest strategy when compared to SOC DOT in Ethiopia and Uganda (1% cheaper in Ethiopia and 6% in Uganda). In India, this strategy was slightly more expensive than SOC DOT, by 0.1%.

### Societal costs

**Fig. 2:**
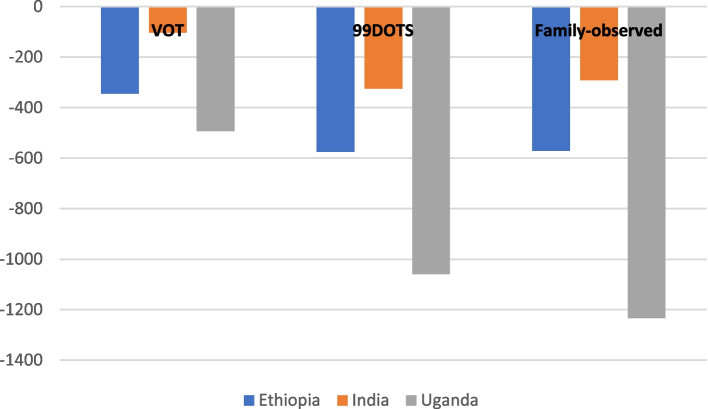
Societal costs of alternative DOT strategies compared to SOC

From a societal perspective, SOC is the costliest approach in all three countries (Fig. 2). This is closely followed by the VOT approach, with savings vs. SOC DOT ranging from 4% in India to 10% in Ethiopia.

Family-observed DOT yields the highest savings vs. SOC DOT from a societal perspective in Uganda, while 99DOTS is the cheapest strategy in Ethiopia and India.

### Sensitivity analyses

Decreasing the LTFU by 5% and 10% made the alternative DOT approaches less costly than in the base case as a consequence of slightly lower health system costs (see supplement). This is because lower patients will need re-treatment.

Results remained robust to an increased relapse rate of 6.5%, although the health system costs for the alternative DOT approaches costs increased (see supplement) as the number of patients needing re-treatment increased.

Findings also remained robust when parameter uncertainty was tested in a probabilistic sensitivity analysis.

## Discussion

This study analyses the potential cost of implementing alternative, more people-centred DOT approaches for MDR-TB patients that follow a 9-month all-oral treatment regimen. The results indicate that use of VOT, 99DOTS and family-observed DOT as part of a 9-month all-oral MDR-TB treatment regimen could result in important societal cost savings and substantially reduce patient costs in all countries. This could protect TB-affected populations from catastrophic expenditure. The results are consistent with other studies [35], which reported societal cost savings of 15% to 18% from the use of alternative DOT approaches, compared to SOC DOT for the long MDR-TB treatment recommended by the WHO in 2011 (now superseded).

SOC DOT requires patients to regularly visit health facilities for DOT, placing a significant cost burden on patients [12] and potentially contributing to LTFU. A qualitative study in Ethiopia reported that traveling long distances to a health facility for SOC DOT generated patient costs that competed with other essential expenses and made it difficult for patients to collect their daily drugs. In that study, patients stated that lack of money for travel to health facilities was the main reason for treatment non-compliance [16]. Other studies reported that patients found SOC DOT inconvenient and preferred VOT over SOC DOT [36, 37]. In contrast, the alternative DOT approaches evaluated in this study permit DOT to take place according to the patient’s circumstances, limiting interruptions to their usual activities while also achieving the same objectives as SOC DOT (i.e., reminding patients to take their medication and/or permitting healthcare workers to monitor treatment adherence). From a health system perspective, VOT and 99DOTS have robust, electronic, data-monitoring systems that can be implemented, possibly making it easier for healthcare workers to monitor treatment adherence and reduce time allocated to this activity [20]. This is in contrast with SOC DOT, which typically uses paper-based treatment cards to record treatment adherence, making data monitoring more time consuming and less efficient.

In the base case model, we assumed that health system costs would remain constant for each new MDR-TB patient, i.e., that mobile phones and data will be bought for all patients who do not own them at treatment initiation. However, VOT and 99DOTS costs could decrease gradually as ownership of mobile phones increases or insurance systems to ensure return of smartphones are put in place. Moreover, some costs, such as renting a toll-free line for 99DOTS or mobile data costs could decline on a per patient basis due to economies of scale as more patients are allocated to the alternative DOT approaches. This would result in additional per patient cost savings for the alternative DOT approaches, when compared to SOC. Additionally, a model similar to the one in the UK [19] could be implemented, where patients pre-record a video while taking the pills and healthcare workers only randomly check 20% of them. This could further reduce health system costs but can also affect treatment adherence.

There was no transmission component included in the model, however, the addition of a transmission model is unlikely to have influenced the findings. First, because previous studies showed that infectiousness of TB patients diminishes rapidly once effective treatment is initiated [38, 39]. Second, because even if transmission was taking place, this would have been the same across all DOT strategies as all patients are treated as outpatients, in line with the local guidelines.

Adopting digital healthcare approaches, thus increasing access to a smartphones and internet connections, may also have benefits beyond DOTs for the patients, such as growing access to education or increasing ease of communication.

This study has a number of limitations. As there is no study assessing the efficacy of the different DOT approaches in the context of shorter MDR-TB regimens, we assumed that DOT strategies would not affect treatment outcomes. Although we tested these assumptions in the sensitivity analyses, more research is needed to understand the efficacy and cost-effectiveness of the alternative DOT strategies, particularly in LMIC countries. Until that research is undertaken, it is difficult to assess the cost-effectiveness of the various DOT approaches presented in this paper. It is possible that these approaches might reduce LTFU and because they are also cheaper, they would be highly likely to be cost-effective compared to SOC DOT. The alternative DOT approaches might also result in more missed doses and thus in worse clinical outcomes, such as increased relapse rates. If this is the case, then the reduced efficacy of alternative DOT strategies might offset their lower cost.

VOT and 99DOTS can only be implemented when the required technology is available and can be appropriately organized and operated by health care providers and patients. This would require patients to have an electricity source to charge their devices (at a minimum). In some countries/populations, this may not be possible for all patients. In those cases, a potential alternative to this is family-observed DOT, which provided substantial societal cost savings in our modelling exercise when compared to SOC DOT.

There are costs that were not captured in the model, including increased utility bills for patients due to higher electricity usage for charging equipment. It also does not include costs related to the training required for patients to use digital technologies, the training required for healthcare workers regarding alternative DOT strategies, or the cost to develop digital treatment monitoring protocols. These are difficult to estimate and would likely differ by country.

### Supplementary Information

**Additional file 1: Table S1.** Unit costs used in calculating health system costs. **Table S2.** Unit costs used in the analysis that were tested in the probabilistic sensitivity analysis. **Table S3.** Scenario analysis where smartphone costs were eliminated from the health system costs. **Table S4.** Scenario analysis for a 6-month all-oral regimen. **Table S5.** Probabilistic sensitivity analysis results. **Table S6.** Deterministic sensitivity analysis on LTFU and relapse rates for the digitally-observed DOT and family-observed DOT. **Figure S1. **Health system costs compared to standard of care for each of VOT, 99DOTS and family-observed DOT, in each country.

## Data Availability

The data used during the current study are publicly available and can be found in the STREAM economic evaluation paper (https://doi.org/10.1016/S2214-109X(22)00498-3), 99DOTS paper (https://www.microsoft.com/en-us/research/uploads/prod/2019/02/99DOTS-ICTD.pdf), Ethio Telecom (https://www.ethiotelecom.et/), MobGSM (https://et.mobgsm.com/mobile/samsung-galaxy-a13-price-in-ethiopia), Airtel (https://www.airtel.in/), Croma (https://www.croma.com/phones-wearables/mobile-phones/c/10) and Jumia (https://www.jumia.ug/). Model probabilities have been calculated using data from the STREAM clinical paper (https://doi.org/10.1016/S0140-6736(22)02078-5). More details are in Table 2 of the main paper and tables S1 and S2 of the supplement.
