# Supplementary material for: Cost of digital technologies and family-observed DOT for a shorter MDR-TB regimen: a modelling study in Ethiopia, India and Uganda
Source: BMC Health Serv Res. 2023 Nov 18;23:1275. doi: 10.1186/s12913-023-10295-z (PMC10657602; doi:10.1186/s12913-023-10295-z)
Supplement: Supplementary file 1 — Additional file 1: Table S1. Unit costs used in calculating health system costs. Table S2. Unit costs used in the analysis that were tested in the probabilistic sensitivity analysis. Table S3. Scenario analysis where smartphone costs were eliminated from the health system costs. Table S4. Scenario analysis for a 6-month all-oral regimen. Table S5. Probabilistic sensitivity analysis results. Table S6. Deterministic sensitivity analysis on LTFU and relapse rates for the digitally-observed DOT and family-observed DOT. Figure S1. Health system costs compared to standard of care for each of VOT, 99DOTS and family-observed DOT, in each country. [file 12913_2023_10295_MOESM1_ESM.docx]

**Supplementary Appendix**

Contents

[2 List of Abbreviatons 2](file:///C:\Users\Laura.Rosu\AppData\Local\Microsoft\Windows\INetCache\Content.Outlook\G79Z7BHV\STREAM%20supplement%202018_08_15%20(003).docx#_Toc521923573)

[3 Listing of supplementary tables](file:///C:\Users\Laura.Rosu\AppData\Local\Microsoft\Windows\INetCache\Content.Outlook\G79Z7BHV\STREAM%20supplement%202018_08_15%20(003).docx#_Toc521923573) 3

[4 Background 4](file:///C:\Users\Laura.Rosu\AppData\Local\Microsoft\Windows\INetCache\Content.Outlook\G79Z7BHV\STREAM%20supplement%202018_08_15%20(003).docx#_Toc521923573)

[5 Detailed Methods](file:///C:\Users\Laura.Rosu\AppData\Local\Microsoft\Windows\INetCache\Content.Outlook\G79Z7BHV\STREAM%20supplement%202018_08_15%20(003).docx#_Toc521923574) 4

[6 Supplementary tables and figures](file:///C:\Users\Laura.Rosu\AppData\Local\Microsoft\Windows\INetCache\Content.Outlook\G79Z7BHV\STREAM%20supplement%202018_08_15%20(003).docx#_Toc521923576) 5

[9 References](file:///C:\Users\Laura.Rosu\AppData\Local\Microsoft\Windows\INetCache\Content.Outlook\G79Z7BHV\STREAM%20supplement%202018_08_15%20(003).docx#_Toc521923599) 9

# List of abbreviations

ALT- Alanine Transaminase

AST- Aspartate Transferase

DOT- Directly-observed treatment

ECG- Electrocardiogram

MDR-TB- Multidrug resistant tuberculosis

SAE- Serious Adverse Event

SOC- Standard of care

STREAM- The Standardised Treatment Regimen of Anti-TB Drugs for Patients with MDR-TB

TB- Tuberculosis

VOT- Video-observed treatment

WHO- World Health Organization

# Listing of Supplementary Tables and Figures

**Table S1**: Unit costs used in calculating health system costs

**Table S2**: Unit costs used in the analysis that were tested in the probabilistic sensitivity analysis

**Table S3**: Scenario analysis where smartphone costs were eliminated from the health system costs

**Table S4**: Scenario analysis for a 6-month all-oral regimen

**Table S5**: Probabilistic sensitivity analysis results

**Table S6**: Deterministic sensitivity analysis on LTFU and relapse rates for the digitally-observed DOT and family-observed DOT

**Figure S1:** Health system costs compared to standard of care for each of VOT, 99DOTS and family-observed DOT, in each country

# Background

WHO recommended the use of digital technologies, such as medication monitors and video observed treatment for directly observed treatment of drug-susceptible TB since 2017. These recommendations were extended with the 2020 guidelines^1^, but without good evidence on either the cost or effects of these for the shorter MDR-TB regimen.

We searched PubMed for studies on digital DOT or family-observed DOT in the context of MDR-TB published from June 2020 when the all-oral, shorter MDR-TB regimen was recommended by WHO to December 2022, with the terms "tuberculosis" AND "rifampicin resistance" OR "rifampicin-resistance" OR "rifampin resistance" OR "rifampin-resistance" OR "MDR" OR "multidrug" OR "multi-drug" OR "MDR-TB" OR "RR-TB" AND "digital health" OR "video observed" OR "video-observed" OR "99DOTS" OR "VOT" OR "video monitoring" OR "message reminders" OR "family DOT" OR "medication monitor" AND "treatment adherence" OR "cure" OR "completion" OR "compliance" OR "cost". This searched yielded 46 results but none of the studies evaluated treatment outcomes or costs of digital DOT or family-observed DOT for the 9-month all-oral regimen; some studies included longer MDR-TB treatment regimens or focused chronic respiratory disease.

Prior to this study, there was evidence that a longer MDR-TB treatment delivered via digital interventions led to cost savings relative to standard of care DOT in Brazil.^1^ Few other studies compared SOC to digital DOT for drug-susceptible TB. ^2,3,4,5,^

Supplementary details of the methods and results presented elsewhere, are reported below.

# Detailed Methods

All costs are reported in 2021 US$.

The local guidelines recommend daily DOT visits in Ethiopia and Uganda and three DOT visits weekly once injectable-containing treatment ended in India.

WHO also recommends that patients attend monthly clinical and safety monitoring visits^6^. As treatment duration was 9-months long, patients had 9 assessment visits where the following tests were done: smear test, culture, ALT, AST, CBC, Serum Creatinine, Serum Potassium, Chest X-ray, and ECG.

Our model also allows for patients who relapse for one re-treatment with the same 9-month treatment and same periodic clinical monitoring visits. Also, our model assumes that patients who are lost to follow-up and not die within one year are re-treated.

70% of people in Uganda^7^, 66% in Ethiopia^8^ and 57% in India^9^ did not own a smartphone in 2021. These penetration rates have been used in calculating the equipment costs for delivering the VOT strategy (see scenario analyses below). Internet connection is also required for making video calls, so we calculated that a 5-minute duration for each VOT visit would require 500MB of data per patient per month and this would be bought for all patients, regardless of whether they own a smartphone or not.^10^ Smartphones and mobile data costs were obtained from phone companies in each country. All unit costs used in the analysis and their sources are in tables S1 and S2.

# Sensitivity analyses

All probabilities were included in the probabilistic sensitivity analysis and a beta distribution was used.

When no information about the parameters was available, as in the case of costs, a distribution was constructed assuming that the 95% credible interval around the mean is represented by the mean +/-30%. Using these credible intervals, a standard error has been calculated and, using the method of moments the parameters for the gamma distribution have been derived (see table S2).

As smartphone ownership is expected to increase in the future, a scenario analysis was also conducted by eliminating the smartphone costs (but not mobile data-related costs) from the health system costs (table S3).

A 6-month all-oral regimen has recently been recommended by WHO for treating MDR-TB. Therefore, a further scenario analysis was conducted to assess how costs would change when treatment duration is reduced. Thus, total number of patient centred visits in Ethiopia and Uganda were assumed to be 180 visits and 60 visits in India. Patients allocated to the health facility DOT would need to make these visits in person. Results are in table S4 and show that the SOC would still be the most expensive strategy in all cases.

Results of the probabilistic sensitivity analysis are in table S5.

# Supplementary tables and figures

Table S1. STREAM unit costs used in calculating health system costs.

| Cost category | Ethiopia (US$) | India (US$) | Uganda (US$) |
| --- | --- | --- | --- |
| Overheads per visit | 0.02 | 0.01 | 0.01 |
| Sputum culture | 7.4 | 2.5 | 5.6 |
| Sputum smear | 7.2 | 5.8 | 16.6 |
| ALT per test | 17.3 | 9.4 | 15.4 |
| AST per test |  |  |  |
| Serum Creatinine per test |  |  | 12.6 |
| Serum Potassium per test |  |  |  |
| Full Blood Count | 3.3 |  | 4.2 |
| ECG per test | 2.7 | 1.6 | 14 |
| TSH&thyroxine of free thyroxine | 5.7 | 5.6 | 14 |
| Chest X-ray | 7.4 | 2 | 8.4 |
| N95 for healthcare worker per item | 1.6 | 2.1 | 1.3 |
| Surgical mask for patients per item | 0.1 | 0.1 | 0.04 |
| Surgical gloves per pair | 0.3 | 0.3 | 0.03 |

Table S2. Unit costs used in the analysis that were tested in the probabilistic sensitivity analysis

|  | Ethiopia | | | India | | | Uganda | | | |
| --- | --- | --- | --- | --- | --- | --- | --- | --- | --- | --- |
| Cost category | Cost (US$) | 95% credible interval | Source | Cost (US$) | 95% credible interval | Source | Cost (US$) | 95% credible interval | Source | Distribution |
| Internet nurse per visit | 0.04 | (0.03, 0.06) | _11_ | 0.4 | (0.3, 0.5) | ^15^ | 0.3 | (0.2, 0.4) | ^17^ | gamma |
| Internet patient per visit | 0.2 | (0.2, 0.3) | _11_ | 0.6 | (0.4, 0.7) | ^15^ | 1.1 | (0.8, 1.5) | ^17^ | gamma |
| Smartphone cost | 234.0 | (163.8, 304.2) | _12_ | 107.3 | (75.1, 139.4) | ^16^ | 155.3 | (108.7, 201.9) | ^17^ | gamma |
| Renting toll free line per treatment duration | 0.03 | (0.02, 0.04) | _13_ | 0.03 | (0.02, 0.04) | _13_ | 0.03 | (0.02, 0.04) | _13_ | gamma |
| Envelopes costs | 2.58 | (1.81, 3.35) | _13_ | 2.58 | (1.81, 3.35) | _13_ | 2.58 | (1.81, 3.35) | _13_ | gamma |
| SMS and call costs | 2.73 | (1.91, 3.55) | _13_ | 2.73 | (1.91, 3.55) | _13_ | 2.73 | (1.91, 3.55) | _13_ | gamma |
| Cost of labor to wrap medication | 0.22 | (0.15, 0.29) | _13_ | 0.22 | (0.15, 0.29) | _13_ | 0.22 | (0.15, 0.29) | _13_ | gamma |
| Indirect cost patient/DOT supervisor per minute | 0.01 | (0.00, 0.01) | ^14^ | 0.01 | (0.01, 0.02) | ^14^ | 0.01 | (0.00, 0.01) | ^14^ | gamma |
| Staff cost per minute | 0.01 | (0.01, 0.01) | ^14^ | 0.05 | (0.04, 0.07) | ^14^ | 0.06 | (0.04, 0.08) | ^14^ | gamma |

Table S3. Scenario analysis where smartphone costs were eliminated from the health system costs

|  | Ethiopia | India | Uganda |
| --- | --- | --- | --- |
| VOT in base case | 3999.917 | 2201.7 | 6716.74 |
| VOT in scenario analysis | 3844.922 | 2140.491 | 6607.643 |
| SOC DOT base case | 3790.36 | 2003.26 | 6348.56 |

Table S4. Scenario analysis for a 6-month all-oral regimen

|  | India (US$) | | | Ethiopia (US$) | | | Uganda (US$) | | |
| --- | --- | --- | --- | --- | --- | --- | --- | --- | --- |
|  | Health system | Patient | Societal | Health system | Patient | Societal | Health system | Patient | Societal |
| SOC | 1965.72 | 198.51 | 2164.23 | 3773.54 | 350.49 | 4,124.03 | 6246.88 | 544.21 | 6791.09 |
| VOT | 2108.06 | 22.67 | 2130.73 | 3965.02 | 17.87 | 3,982.88 | 6517.53 | 27.74 | 6545.27 |
| 99DOTS | 1956.28 | 22.11 | 1978.39 | 3769.34 | 17.90 | 3,787.24 | 6141.06 | 27.43 | 6168.49 |
| Family-observed | 1968.79 | 31.76 | 2000.55 | 3765.41 | 26.33 | 3,791.74 | 5974.96 | 29.48 | 6004.44 |

Table S5. Probabilistic sensitivity analysis results

|  | Ethiopia (US$) | | | India (US$) | | | Uganda (US$) | | |
| --- | --- | --- | --- | --- | --- | --- | --- | --- | --- |
|  | Health system | Patient | Societal | Health system | Patient | Societal | Health system | Patient | Societal |
| SOC | 3732.41 | 570.16 | 4362.62 | 1899.26 | 322.95 | 2327.41 | 6095.52 | 885.29 | 7237.13 |
| VOT | 3901.62 | 17.80 | 3919.42 | 1997.39 | 22.59 | 2019.98 | 6499.54 | 27.61 | 6749.48 |
| 99DOTS | 3754.11 | 17.83 | 3771.94 | 1912.85 | 22.03 | 1934.88 | 6121.71 | 27.33 | 6183.73 |
| Family-observed | 3748.61 | 6.49 | 3755.11 | 1907.52 | 20.83 | 1928.35 | 5937.23 | 16.32 | 6009.49 |

Table S6. Deterministic sensitivity analysis on LTFU and relapse rates for the digitally-observed and family-observed DOT

1. A 5% LTFU rate was tested

|  | Ethiopia (US$) | | | India (US$) | | | Uganda (US$) | | |
| --- | --- | --- | --- | --- | --- | --- | --- | --- | --- |
| LTFU- 5% | Health system | Patient | Societal | Health system | Patient | Societal | Health system | Patient | Societal |
| SOC | 3790.4 | 572.3 | 4362.6 | 2003.3 | 324.1 | 2327.4 | 6348.6 | 888.6 | 7237.1 |
| VOT | 3996.6 | 17.9 | 4014.5 | 2200.1 | 22.7 | 2222.8 | 6711.2 | 27.7 | 6738.9 |
| 99DOTS | 3766.2 | 17.9 | 3784.0 | 1978.7 | 22.1 | 2000.8 | 6146.0 | 27.4 | 6173.4 |
| Family-observed | 3762.2 | 26.3 | 3788.5 | 2003.3 | 31.7 | 2035.0 | 5977.4 | 29.5 | 6006.9 |

1. A 10% LTFU rate was tested

|  | Ethiopia (US$) | | | India (US$) | | | Uganda (US$) | | |
| --- | --- | --- | --- | --- | --- | --- | --- | --- | --- |
| LTFU- 10% | Health system | Patient | Societal | Health system | Patient | Societal | Health system | Patient | Societal |
| SOC | 3790.4 | 572.3 | 4362.6 | 2003.3 | 324.1 | 2327.4 | 6348.6 | 888.6 | 7237.1 |
| VOT | 3993.3 | 17.8 | 4011.2 | 2198.6 | 22.6 | 2221.2 | 6705.7 | 27.7 | 6733.4 |
| 99DOTS | 3763.0 | 17.9 | 3780.8 | 1977.1 | 22.1 | 1999.1 | 6140.8 | 27.4 | 6168.2 |
| Family-observed | 3759.1 | 26.3 | 3785.3 | 2001.6 | 31.7 | 2033.3 | 5974.9 | 29.4 | 6004.3 |

1. A 6.5% relapse rate

|  | Ethiopia (US$) | | | India (US$) | | | Uganda (US$) | | |
| --- | --- | --- | --- | --- | --- | --- | --- | --- | --- |
| Relapse 6.5% | Health system | Patient | Societal | Health system | Patient | Societal | Health system | Patient | Societal |
| SOC | 3790.4 | 572.3 | 4362.6 | 2003.3 | 324.1 | 2327.4 | 6348.6 | 888.6 | 7237.1 |
| VOT | 4002.9 | 17.9 | 4020.8 | 2203.3 | 22.7 | 2226.0 | 6721.7 | 27.8 | 6749.5 |
| 99DOTS | 3772.4 | 17.9 | 3790.4 | 1982.0 | 22.1 | 2004.2 | 6156.3 | 27.5 | 6183.7 |
| Family-observed | 3768.5 | 26.4 | 3794.9 | 2006.6 | 31.8 | 2038.4 | 5980.0 | 29.5 | 6009.5 |

Figure S1. Health system costs compared to standard of care for each of VOT, 99DOTS and family-observed DOT, in each country

# References

1. World Health Organization. WHO consolidated guidelines on tuberculosis. Module 4: treatment-drug-resistant tuberculosis treatment. World Health Organization 2020.
2. Nsengiyumva, N.P., Mappin-Kasirer, B., Oxlade, O., Bastos, M., Trajman, A., Falzon, D. and Schwartzman, K., 2018. Evaluating the potential costs and impact of digital health technologies for tuberculosis treatment support. *European Respiratory Journal*, *52*(5).
3. Lund, S., Hemed, M., Nielsen, B.B., Said, A., Said, K., Makungu, M.H. and Rasch, V., 2012. Mobile phones as a health communication tool to improve skilled attendance at delivery in Zanzibar: a cluster‐randomised controlled trial. *BJOG: An International Journal of Obstetrics & Gynaecology*, *119*(10), pp.1256-1264.
4. Liu, X., Lewis, J.J., Zhang, H., Lu, W., Zhang, S., Zheng, G., Bai, L., Li, J., Li, X., Chen, H. and Liu, M., 2015. Effectiveness of electronic reminders to improve medication adherence in tuberculosis patients: a cluster-randomised trial. *PLoS medicine*, *12*(9), p.e1001876.
5. Chuck, C., Robinson, E., Macaraig, M., Alexander, M. and Burzynski, J., 2016. Enhancing management of tuberculosis treatment with video directly observed therapy in New York City. *The International Journal of Tuberculosis and Lung Disease*, *20*(5), pp.588-593.
6. Fang, X.H., Guan, S.Y., Tang, L., Tao, F.B., Zou, Z., Wang, J.X., Kan, X.H., Wang, Q.Z., Zhang, Z.P., Cao, H. and Ma, D.C., 2017. Effect of short message service on management of pulmonary tuberculosis patients in Anhui Province, China: a prospective, randomized, controlled study. *Medical science monitor: international medical journal of experimental and clinical research*, *23*, p.2465.
7. World Health Organization. A people-centred model of tuberculosis care. A blueprint for eastern European and central Asian countries, first edition. World Health Organization 2017.
8. Uganda Communications Commission, Market Performance Report, 2021. Available at: <https://www.ucc.co.ug/wp-content/uploads/2021/07/UCC_1Q21-MARKET-PERFOMANCE-REPORT_-compressed.pdf> [Accessed 28.02.2023]
9. GSM Association, The Mobile Economy Sub-Saharan Africa, 2021. Available at: <https://www.gsma.com/mobileeconomy/wp-content/uploads/2021/09/GSMA_ME_SSA_2021_English_Web_Singles.pdf> [Accessed 29.12.2022]
10. List of countries by smartphone penetration, Wikipedia, Available at: <https://en.wikipedia.org/wiki/List_of_countries_by_smartphone_penetration> [Accessed 29.12.2022]
11. Make Tech Easier, How much bandwidth does video calling use? Available at: <https://www.maketecheasier.com/how-much-bandwidth-does-video-calling-use/> [Accessed 29.12.2022]
12. Ethio Telecom, Ethiopia. Available at: <https://www.ethiotelecom.et/> [Accessed 28.03.2022]
13. MobGSM, Ethiopia. Available at: <https://et.mobgsm.com/mobile/samsung-galaxy-a13-price-in-ethiopia> [Accessed 28.03.2022]
14. Cross, A., Gupta, N., Liu, B., Nair, V., Kumar, A., Kuttan, R., Ivatury, P., Chen, A., Lakshman, K., Rodrigues, R. and D'Souza, G., 2019, January. 99DOTS: a low-cost approach to monitoring and improving medication adherence. In *Proceedings of the Tenth International Conference on Information and Communication Technologies and Development* (pp. 1-12).
15. Rosu, L., Madan, J.J., Tomeny, E.M., Muniyandi, M., Nidoi, J., Girma, M., Vilc, V., Bindroo, P., Dhandhukiya, R., Bayissa, A.K. and Meressa, D., 2023. Economic evaluation of shortened, bedaquiline-containing treatment regimens for rifampicin-resistant tuberculosis (STREAM stage 2): a within-trial analysis of a randomised controlled trial. *The Lancet Global Health*, *11*(2), pp.e265-e277.
16. Airtel, India. Available at: <https://www.airtel.in/> [Accessed 28.03.2022]
17. Croma, India. Available at: <https://www.croma.com/phones-wearables/mobile-phones/c/10> [Accessed 28.03.2022]
18. Jumia, Uganda. Available at: <https://www.jumia.ug/> [Accessed 28.03.2022]
